# Modelling the burden of hepatitis C infection among people who inject drugs in Norway, 1973–2030

**DOI:** 10.1186/s12879-017-2631-2

**Published:** 2017-08-03

**Authors:** Hinta Meijerink, Richard A White, Astrid Løvlie, Birgitte Freiesleben de Blasio, Olav Dalgard, Ellen J. Amundsen, Espen Melum, Hilde Kløvstad

**Affiliations:** 1 Norwegian Institute of Public Health, Postboks 4404 Nydalen, 0403 Oslo, Norway; 2 European Programme for Intervention Epidemiology Training (EPIET), European Centre for Disease Prevention and Control, (ECDC), Stockholm, Sweden; 3 Department of Biostatistics, Oslo Centre for Biostatistics and Epidemiology, Institute of Basic Medical Sciences, University of Oslo, Oslo, Norway; 4 Akershus University Hospital, Lørenskog, Norway; 5 Medical Faculty, Oslo University, Oslo, Norway; 6 Norwegian PSC Research Center, Division of Surgery, Inflammatory Medicine and Transplantation, Oslo University Hospital, Rikshospitalet, Oslo, Norway; 7 Section of Gastroenterology, Division of Surgery, Inflammatory Medicine and Transplantation, Oslo University Hospital, Rikshospitalet, Oslo, Norway; 8 Research Institute of Internal Medicine, Division of Surgery, Inflammatory Medicine and Transplantation, Oslo University Hospital, Rikshospitalet, Oslo, Norway

**Keywords:** Burden of disease, Hepatitis C, Intravenous substance abuse, Natural history of disease model, People who inject drugs, Norway

## Abstract

**Background:**

Lack of Hepatitis C virus (HCV) incidence data in (Norwegian) high-risk groups impedes the ability to make informed decisions on prevention measures. Thus we rely on modelling to estimate the incidence and burden of HCV infections.

**Methods:**

We constructed a compartmental model for HCV infections in Norway among active and former people who inject drugs (PWIDs). We based yearly transition rates on literature. The model was fitted to absolute numbers of hepatitis C associated cirrhosis, hepatocellular carcinoma (HCC) and death from national data sources (2000–2013). We estimated the number (95%CI) of HCV infections, cirrhosis, HCC and death and disability adjusted life years (DALYs) due to HCV infections in Norway, 1973–2030. We assumed treatment rates in the projected period were similar to those in 2013.

**Results:**

The estimated proportion of chronic HCV (including those with cirrhosis and HCC) among PWIDs was stable from 2000 (49%; 4441/9108) to 2013 (43%; 3667/8587). We estimated that the incidence of HCV among PWIDs was 381 new infections in 2015. The estimated number of people with cirrhosis, HCC, and liver transplant was predicted to increase until 2022 (1537 people). DALYs among active PWIDs estimated to peak in 2006 (3480 DALYs) and decrease to 1870 DALYs in 2030. Chronic HCV infection contributes most to the total burden of HCV infection, and peaks at 1917 DALYs (52%) in 2007. The burden of HCV related to PWID increased until 2006 with 81/100,000 DALYs inhabitants and decreased to 68/100,000 DALYs in 2015.

**Conclusion:**

The burden of HCV associated with injecting drug use is considerable, with chronic HCV infection contributing most to the total burden. This model can be used to estimate the impact of different interventions on the HCV burden in Norway and to perform cost-benefit analyses of various public health measures.

**Electronic supplementary material:**

The online version of this article (doi:10.1186/s12879-017-2631-2) contains supplementary material, which is available to authorized users.

## Background

Worldwide, an estimated 71.1 (62.5–79.4) million people are chronically infected with hepatitis C virus (HCV) [1–3]. Untreated, 7–18% of those infected progress to liver disease within 20 years, such as cirrhosis, hepatocellular carcinoma (HCC) and liver failure, which can subsequently lead to death [4]. An estimated 500,000 people die from HCV related liver diseases yearly [2]. It is difficult to clearly define the natural history of HCV infection due to the long course of the disease. The 20-year cumulative probability of cirrhosis, as estimated by fibrosis progression rates obtained from a large number of studies, was 16% (95% CI: 14–19%) [5] and the risk of developing HCC once cirrhosis developed has been estimated to be up to 3% annually [6, 7]. At the moment, liver transplantation (LTX) is the only curative treatment for end stage liver disease caused by HCV [8].

Surveillance of HCV is challenging for several reasons. Infection can be asymptomatic for many years [4] and therefore many people may only become aware during routine screening of risk groups, when a blood test reveals increased liver function tests or when symptoms arise; some will even progress through life without ever developing symptoms [4]. Few people present with an acute stage of HCV and current laboratory tests cannot distinguish between acute and chronic HCV infection, making incidence estimation difficult. In addition, people who inject drugs (PWIDs), a major risk group for HCV, may be less likely to seek health care [9, 10].

The prevalence of HCV infection in the general population in Norway is low. HCV antibodies (indicating current infection, clearance or successful treatment of HCV infection) were detected in 0.8% of the Norwegian population in 2000–2001 [11]. Current infection, identified by HCV-RNA, was present in 0.5% of the population [11]. Like in most developed countries, the primary mode of HCV transmission in Norway is through sharing of needles, syringes and drug injection paraphernalia amongst PWIDs. However, immigrants from high-incidence countries account for a considerable number of patients with liver disease and those infections are often not related to drug use. In 85% of all HCV cases notified to the Norwegian Surveillance System for Communicable Diseases (MSIS) injecting drug use was the suspected route of transmission [12].

In Norway, HCV antibody tests have been available since 1990. Notification of acute HCV infection (based on clinical symptoms and positive HCV antibody test) has been mandatory since 1992. From 2008 onwards, all HCV positive cases were notifiable to MSIS by both clinicians and laboratories [12]. No distinction is made between acute and chronic HCV infection and there is considerable uncertainty about the current burden of hepatitis C in Norway. The case definition for reporting HCV cases was changed in 2016 to include only HCV RNA positive and/or HCV core antigen positive.

Interferon (IFN)-based treatment regimens have been the standard of care for chronic HCV infection. These therapies are associated with insufficient response and frequent side effects, resulting in a limited number of patients treated. With the recent introduction of direct-acting antivirals (DAAs) higher treatment success, fewer side effects, and more simple regimens can be expected. A range of different drugs are currently available; according to the Norwegian Prescription Database, at least 11 of these are in use Norway: ribavirin, daclatasvir, sofosbuvir, dasabuvir, ledipasvir, ombitasvir, paritaprevir, ritonavir and peginterferon alpha-2a. Randomized controlled trials (RCTs) have reported differences in efficacy between drugs, combinations of drugs and treatment durations. The introduction of DAAs has also resulted in a considerable increase in expenditures, with drug costs being the major issue [13–16]. For instance, costs in Norway were more than double from the period before the first DAA (boceprevir) was introduced to the year after. Later, when more DAAs were introduced, expenditures skyrocketed with drug costs increasing tenfold from 2013 to 2015, from €6 million to €61 million (www.reseptregisteret.no). In 2016, the Norwegian government successfully carried out their first tender on hepatitis C drugs, which resulted in price rebates up to 50% [17]. More patients will be eligible for treatment, but higher prices may restrict wide usage. A burden of disease model for HCV among PWIDs will enable us to estimate the impact of implementing such new regimes.

The aim of the present study is to employ mathematical modelling to estimate the incidence and disease burden of HCV among active and former PWIDs in Norway, in order to provide important information needed to prioritize public health measures for HCV in Norway.

## Methods

### Markov model

**Fig. 1:**
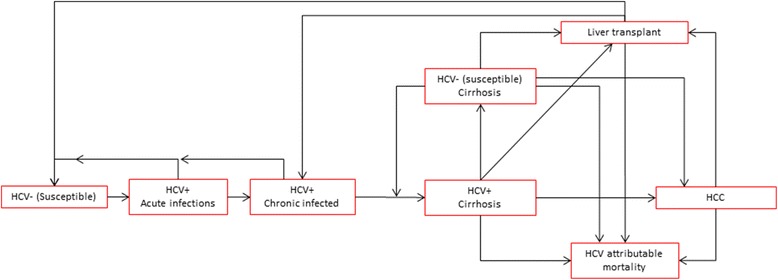
Compartmental model of hepatitis C (HCV) progression among people who inject drugs in Norway. *HCV: hepatitis C; HCC: hepatocellular carcinoma*

We constructed a Markov model for the natural history model of HCV, which follows the disease dynamics in a population over time, in former and current PWID including compartments with eight different HCV-related health states (HCV negative susceptible, acute HCV infection, chronic HCV infection, HCV positive and cirrhosis, HCV negative and cirrhosis, HCC, LTX, HCV related mortality; Fig. 1). The annual transition probabilities between compartments were estimated using a numerical optimizer [18] (details further in supplementary material) where the probability estimates were restricted to be within bounds determined by previous estimates in the literature (Table 1).

**Table 1:** Summary of main data sources for the model

	Source
*Used to hardcode parts of the model*	
Infectivity of PWIDs	The infectivity of PWIDs was modulated by a) the proportion of PWIDs who were infectious, b) the dispersion of the injecting epidemic throughout Norway (the more disperse, the less infectious) (dispersion over time is shown in Additional file 1: Figure S1), and c) the coverage of needle and syringe programs (Additional file 1: Figure S1).
People who inject drugs	Norwegian Institute for Alcohol and Drug Research (SIRUS) has non-internally consistent numbers of new PWIDs and prevalence of active PWIDs, former PWIDs who will relapse, and former PWIDs who will never relapse for each year from 1973 to 2013. We subsequently estimated internally consistent PWID numbers from 1973 to 2030 (Additional file 1: Figure S2).
Age of injecting debut	Age of injecting debut was taken from SIRUS estimates in 1975, 1985, and 1995, and the life quality report from 2003 to 2012. We predicted mean age of injecting debut from 1973 to 2030 using linear regression (Additional file 1: Figure S3).
*Model Targets*	
PWIDs with HCV	The proportion of PWIDs with HCV RNA was based on data collected through cross sectional health studies among PWIDs attending low threshold harm reduction-based health care centers in Oslo targeted towards drug users [12].
Cirrhosis	We received data from one hospital (Akershus universitetssykehus HF) on the number of PWIDs (current and former) treated with cirrhosis associated with HCV in 2013. We extrapolated the total number of cases treated in Norway by dividing by the hospital’s catchment area (10%).
HCC disease	The number of liver cancer or HCC cases were obtained from the Norwegian cancer registry (NCR) using ICD10 code C22 (Malignant neoplasm of liver and intrahepatic bile ducts, which includes HCC). Numbers were adjusted by the proportion of HCC among those with ICD10 code C22 (77%) and for disease attributable to HCV in Norway (26%), resulting in 20% of the extracted data estimated to be HCV associated HCC [25].
Liver transplants	From the Nordic Liver Transplant Registry (NLTR) [21] we used the number of all liver transplants performed from 2000 to 2013 with antibodies against HCV as targets for liver transplants in the model. Mortality within the first year after the LTX is considered as HCV related mortality. After the first year the individuals who received a LTX have the same risk as the rest of the population in the model.
Cirrhosis mortality	Aggregate data on death entries (2000–2013) were obtained from the “Norwegian Cause of Death Registry”. We included individuals with an underlying cause of cirrhosis (ICD10 codes K74.3, K74.4, K74.5 and K74.6). The mortality numbers were then adjusted to the attributable risk of hepatitis C (14%) [26].
HCC mortality	Aggregate data on death entries (2000–2013) were obtained from the “Cause of Death Registry”. We included individuals with an underlying cause of HCC (ICD10 code C22). The mortality numbers were adjusted for HCC among those with ICD10 code C22 (77%) and for to the attributable risk of hepatitis C (26%) [25]
Treatment rates	The Norwegian prescription database (NorPD) was used to estimate the treatment rates for HCV in Norway. Genotype distribution of HCV was based on data from the NIPH [12]. We estimated treatment success for each year, combining the absolute number of treatments from NorPD with treatment response and duration of treatment per genotype.
*Used to calculate health estimates from output*	
Disability weightings	Using the Global Burden of Disease study 2010 disability weights [23], we assigned disability weights to the various health stages in the model.

The first compartment contains HCV negative, susceptible, current PWIDs, which has an influx of newly started PWIDs (Fig. 1). Among active PWIDs, there is a risk of contracting HCV and transfer to the compartment with acute HCV infection. These people will either clear infection and return to the HCV negative (susceptible) group or develop a chronic HCV infection. Results from studies of reinfection risk after spontaneous clearance in PWIDs are conflicting [19] and we therefore choose to consider the risk of reinfection the same as risk of infection. People are classified as having chronic infection if the HCV infection is not spontaneously cleared within one year and if they have not developed cirrhosis. As we are focused on HCV infections associated with drug use, we assume that former PWIDs have no risk of acquiring HCV infection, unless they resume injecting drugs and return to the active PWIDs part of the model. Individuals with chronic infection can develop cirrhosis and subsequently HCC. Former PWIDs with cirrhosis and/or HCC can receive treatment for the liver disease by getting a LTX. Individuals can die from HCV related causes after developing cirrhosis, HCC or after LTX. Individuals receiving a LTX have an increased risk of mortality for the following year, mainly related to surgical and early post-operative complications. HCV related mortality in individuals with cirrhosis is reduced by a factor of five after successfully receiving HCV treatment and becoming HCV negative [20].

Individuals with chronic HCV and chronic HCV with cirrhosis can receive HCV treatment, if successful they return to the respective HCV negative, susceptible compartment. Age-specific mortality is taken into account, including additional risk of mortality for PWIDs (Table 1). The model is replicated for active PWIDs, former PWIDs who will relapse, and former PWIDs who will never relapse. In all stages of the disease individuals can either stop injecting drugs (i.e. transfer from active PWIDs to former PWIDs) or start injecting drugs (transfer from former PWIDs who will relapse to active PWIDs) or stop with injecting completely (transfer from PWIDs to former PWIDs who will never relapse). In addition, the model considers HCV infection with three different genotypes that are assumed to have distinct response rates to treatment. We assumed treatment rates in the projected period were similar to those in 2013. The model is age-stratified into one-year age groups (1–100 years).

### Infectiousness

Baseline infectiousness (“pold” in the supplemental methods, and S - > HCV_AI in Table 2) was estimated alongside the other parameters and was assumed to be constant throughout the study period (1973 to 2030). However, to reflect reality, “actual infectiousness” (i.e. the actual probability of a susceptible person being infected with HCV in year X) was calculated each year from baseline infectiousness modulated by a) the proportion of PWIDs who were infectious (i.e. the more people around you with a disease, the more likely you are to be infected), b) the dispersion of the injecting epidemic throughout Norway (the more disperse, the less infectious) (Additional file 1: Figure S1), and c) the coverage of needle and syringe programs (Additional file 1: Figure S1).

**Table 2:** Parameters used to estimate hepatitis C burden among people who injecting drugs in Norway, 1973–2030

	Estimate			From literature			
Variable	Estimate	Lower	Upper	Lower	Upper	Estimated^b^	Data source^b^
*Injecting drug parameters*							
Excess PWID mortality	0.022	-	-	-	-	No	[28]
Yearly probability of ex-PWID relapse	0.116	-	-	-	-	Yes	Internal SIRUS estimates
Yearly probability of PWID temporary cessation	0.114	-	-	-	-	Yes	Internal SIRUS estimates
Yearly probability of PWID permanent cessation	0.025	-	-	-	-	No	Internal SIRUS estimates
*Genotype prevalence*							
Genotype 1	0.350	-	-	-	-	No	MSIS
Genotype 2	0.150	-	-	-	-	No	MSIS
Genotype 3	0.500	-	-	-	-	No	MSIS
Probability of successful treatment							
Genotype 1	0.450	-	-	-	-	No	[29]
Genotype 2	0.800	-	-	-	-	No	[29]
Genotype 3	0.800	-	-	-	-	No	[29]
*Time-dependent changes to yearly transition probabilities*							
Yearly multiplicative change in treatment from 2004	1.073	1.065	1.092	1.001	1.100	Yes	Expert opinion
Proportion of infectiousness reduction with 100% needle exchange coverage^a^	0.316	0.206	0.356	0.001	0.500	Yes	Expert opinion
Yearly multiplicative change in LT from 2000	1.232	1.166	1.284	1.001	1.300	Yes	Expert opinion
*Yearly transition probabilities*							
S - > HCV_AI^c^	0.083	0.081	0.085	0.010	0.400	Yes	Expert opinion
HCV_AI - > HCV_CI	0.733	0.721	0.766	0.710	0.860	Yes	[30]
HCV_CI - > T_CI	0.046	0.041	0.050	0.020	0.080	Yes	[31]
HCV_CI - > HCV_C	0.014	0.014	0.015	0.001	0.052	Yes	[29]
HCV_C - > T_C	0.306	0.297	0.338	0.010	0.400	Yes	Expert opinion
HCV_C - > HCC	0.021	0.019	0.021	0.014	0.084	Yes	[29, 32, 33]
HCV_C - > LT	0.001	0.001	0.006	0.000	0.020	Yes	Expert opinion
HCV_C - > M	0.034	0.032	0.035	0.025	0.035	Yes	[32, 33]
HCC - > LT	0.056	0.031	0.056	0.012	0.056	Yes	[29]
HCC - > M	0.555	0.547	0.628	0.545	0.676	Yes	[32]
LT - > HCV_CI	0.326	0.116	0.485	0.050	0.500	Yes	Expert opinion
LT - > M	0.165	0.126	0.177	0.110	0.180	Yes	[32]

^a^Infectiousness = Infectiousness^b^(1-InfectiousnessReduction^b^NeedleCoverage)

^b^The “Estimated” column reflects whether or not the estimates are calibrated parameters or taken directly from the literature. The “Data source” column reflects either the literature point estimates (if estimated = no) or the literature upper and lower bounds used in parameter estimation (if estimated = yes)

^c^See the “Infectiousness” section in the methods section for more details

*HCV: hepatitis C infection, PWID: people who inject drugs, S: susceptible, AI: acute HCV infection, CI: chronic HCV infection, T: treatment, C: cirrhosis, HCC: hepatocellular carcinoma, LT: liver transplant, M: HCV associated mortality*

With respect to the dispersion of the injecting epidemic, we used the annual number of drug-related (overdoses) deaths in each Norwegian county as a proxy for the size of the injecting epidemic (e.g. number of PWIDs) in those regions and then calculated the Gini coefficient. If all drug deaths occurred in one county, then the Gini coefficient would be one, and the infectivity high. As drug deaths become more equally distributed throughout the country, the Gini coefficient drops, and so does infectivity (e.g. it is much easier for a PWID in Oslo to infect another PWID in Oslo than it is to infect a PWID in the Northern part of the country). The Gini coefficient variable was included to damper the homogeneous mixing assumption without adding too much complexity to the model. Without the Gini coefficient variable the percentage of PWIDs with HCV was consistently overestimated by 15–20 percentage points.

### Data sources and parameter estimation

**Fig. 2:**
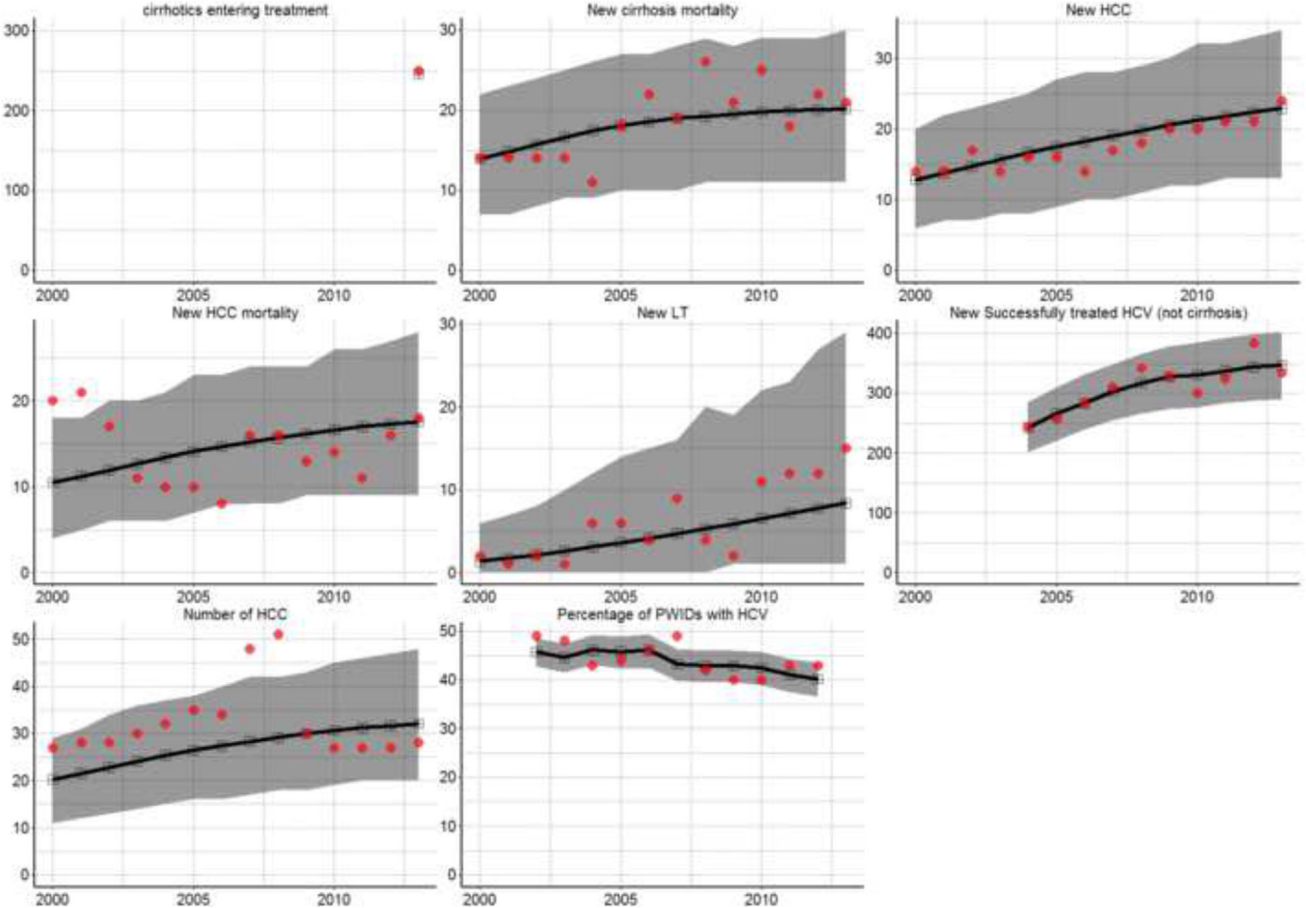
Fitted estimated number (line) with reported numbers (dots) for various health states in Norway, 1973–2030. *Health states: Hepatitis C (HCV) infection, cirrhosis, hepatocellular carcinoma (HCC), liver transplant (LT) and HCV associated mortality. Grey shaded area shows 95% confidence interval*

We based yearly transition rates on literature, MSIS for genotype distribution, the Norwegian Cause of Death Registry for mortality rates, and Norwegian Institute for Alcohol and Drug Research (SIRUS) for entry and exit rates among IDU. In the model we have taken into account the spread of PWID in Norway based on the annual number of drug-related deaths in each Norwegian county (for detailed information see supplementary material). The model was fitted to absolute annual numbers of PWIDs with HCV, incident number of cirrhosis patients under treatment, incident number of liver cancer and/or HCC patients under treatment, incident number of liver transplants, incident number of cirrhosis mortality, incident number of HCC mortality, and incident number of HCV treatment successes (Fig. 2). These target numbers were obtained using a combination of data from SIRUS, National Patient Registry (NPR), Norwegian Cancer Registry (NCR), Nordic Liver Transplant Registry (NLTR) [21] and Norwegian Cause of Death Registry. More detailed information can be found in Table 1.

Given a particular set of transition parameters, we simulated the model from 1973 (the start of the injecting epidemic in Norway) to 2030. We used a numerical optimizer [18] to simultaneously find the set of transition parameters that gave the smallest weighted least-squares error when comparing model estimates to observed data (Fig. 2). The transition probability boundaries were specified using literature and data from Norwegian registries (Table 2). After transition probabilities were estimated, confidence intervals for each probability were calculated using a likelihood ratio test. Subsequently, 1000 random draws of each transition probability were drawn from a random beta distribution, calibrated using the methods of moments. For each of these 1000 draws, the model was run and estimates for incidence, prevalence, years of life lost (YLLs) due to premature death, years lived with disabilities (YLDs) and disability adjusted life years (DALYs) were calculated. In this paper, the burden of disease is given as DALYs or DALYs per 100,000. The 2.5th and 97.5th percentiles were saved as the 95% confidence interval (CI). Model fit to observed data was evaluated visually for each variable (Additional file 1: Figure S3). The model was implemented in R (software available at https://www.r-project.org/). More detailed methods are available in the supplemental materials (Additional file 2).

Sensitivity analyses were conducted by rerunning the model with the transition probabilities set to the maximum and minimum values as specified by the medical literature. We then compared the estimated prevalences in 2015 to the estimates from the final fitted model.

## Results

When comparing our estimates to our known observed data we found that our estimates generally passed through the centre masses of the respective variables, with no obvious bias or tendency to over or under estimate (Fig. 2). The vast majority of the observed data fell within our 95% confidence intervals and we only failed to capture a few extreme observations that were not congruent with the rest of the data (e.g. excessive number of HCC cases in 2007 and 2008).

**Fig. 3:**
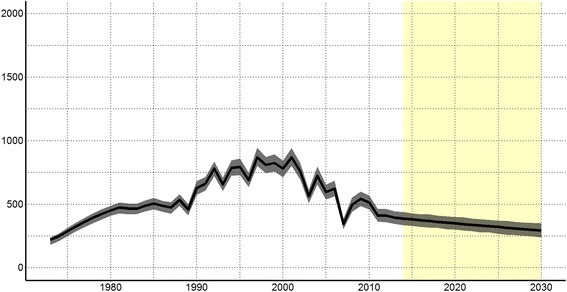
Estimated number of new hepatitis C infections among people who inject drugs in Norway 1973–2030. *HCV: hepatitis C, PWID: people who inject drugs. 95% confidence interval are shaded*

We estimated the number of newly infected PWIDs, using the number of individuals that transition from susceptible to acute HCV infection each year (Fig. 3). Since the start of injecting drug use in Norway in 1973, we see an increase in number of newly HCV infected PWIDs, mostly due to increasing numbers of active PWIDs (Fig. 3). After 2000 the number of newly HCV infected PWIDs decreased to 396 (95%CI: 346–443) (8%; 46 new infections per 1000 active PWIDs) in 2013. In 2015, the estimated incidence of HCV was 7.6% (381 new infections; 44 new infections per 1000 active PWIDs).

**Fig. 4:**
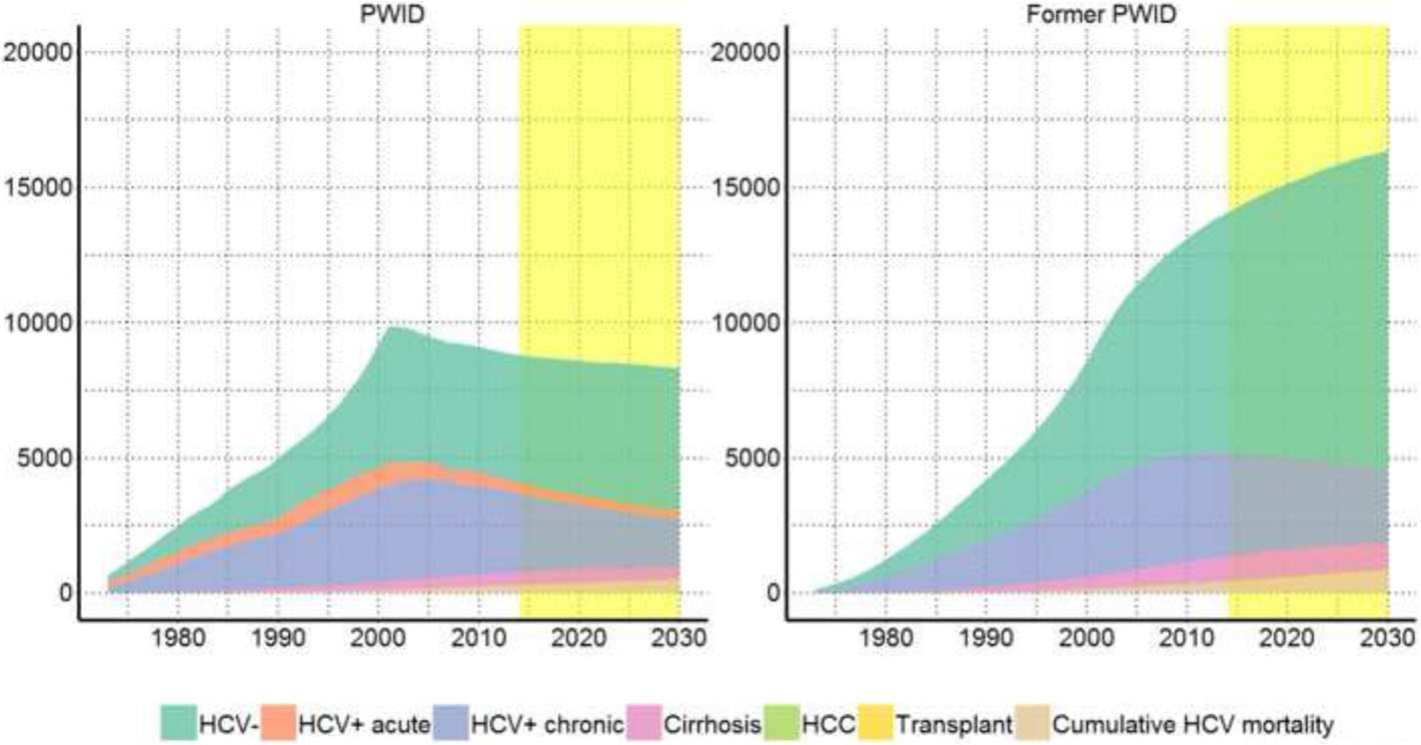
Number of people in various health states among people who inject drugs in Norway, 1973–2030. *Hepatitis C: HCV, PWID: people who inject drugs. Left shows active and right shows former PWIDs. Health states: HCV negative in dark green, acute HCV in orange, chronic HCV in purple, cirrhosis in pink, hepatocellular carcinoma in light green, received a liver transplants in yellow and cumulative mortality related to HCV*. *Yellow shaded area shows predictions*

The total estimated number of active PWIDs (Fig. 4a) increased from 1973 to 2002, then decreased and stabilised at around 9000 active PWIDs (estimated 8587 PWIDs in 2013) (Fig. 4). Number of former PWIDs has been increasing, with the steepest increase up to 2005. The estimated proportion of chronic HCV among PWIDs (including those with cirrhosis and HCC) has been moderately stable from 2000 (48.8% = 4441/9108) to 2013 (42.7% = 3667/8587).

The estimated number of people living with cirrhosis and HCC attributable to HCV was predicted to increase until 2022 (1537 people) (Fig. 4). Decrease of the total numbers was mostly due to a decrease in number of newly infected PWIDs after 2002. The total number of yearly deaths due to HCV was predicted to monotonically increase, reaching 40 deaths in 2015 (Fig. 4). The sensitivity analyses showed that the infection rate and treatment rate were the two largest impactors on HCV+ chronic and cirrhosis prevalence in 2015 (Additional file 1: Figure S4).

The estimated total HCV burden related to injecting drug use was 67.71 DALYs per 100,000 inhabitants in Norway in 2015. The DALYs among active PWIDs were estimated to peak in 2006 with 3480 DALYs (3779/10,000 PWIDs) and to decrease to 1870 DALYs (2390/10,000 PWIDs) in 2030 (Table 3). The DALYs among former PWIDs was estimated to peak in 2011 with 4181 DALYs (3203/10,000 former PWIDs) and to decrease to 3005 DALYs (1935/10,000 former PWIDs) in 2030 (Table 3).

**Table 3:** Estimates from the model on hepatitis C burden among people who inject drugs in Norway

	Absolute numbers with 95% CI						% attribution PWID^a^		
	2000		2015		2030		2000	2015	2030
*Overlapping prevalence*									
Alive	17,613	17,069–17,554	22,292	2159–22,243	23,355	22,584–23,344	52	38	34
HCV+ (incl. Treatment)	7911	7292–7885	7682	6901–7686	5552	4801–5566	56	45	42
HCV+ (excl. Treatment)	7525	6935–7498	6892	6150–6904	4657	3968–4684	57	46	43
HCV treatment	387	333–382	790	669–784	895	747–889	47	40	35
*Discrete prevalence*									
HCV+ chronic	6622	6066–6596	6549	5894–6550	4486	3839–4496	52	43	40
Cirrhosis	771	635–758	1421	1163–1642	1419	1017–1444	39	34	30
HCC	20	11–20	33	19–32	27	16–28	40	39	37
Transplant	1	0–1	10	2–7	13	4–11	0	10	8
*Incidence*									
HCV+ acute	781	713–780	381	330–380	294	240–296	100	100	100
HCV+ chronic	605	541–606	285	239–285	219	176–220	86	86	86
Cirrhosis	86	66–86	88	66–88	58	41–58	49	41	38
HCC	13	6–12	24	14–24	23	13–23	38	33	30
Transplant	1	0–1	10	2–7	13	4–11	0	10	8
*Yearly mortality*									
Cirrhosis	14	7–14	20	12–20	18	10–18	43	35	33
HCC	11	4–10	18	10–18	15	8–16	36	39	40
Transplant	0	0–0	1	0–1	2	0–2		0	0
Total HCV related	25	15–24	40	27–40	36	24–36	40	35	33
Total not related-HCV	243	213–242	256	224–256	354	311–352	96	85	64
*Cumulative mortality*									
Total HCV related	243	189–235	764	639–753	1345	1151–1337	44	40	38
Total not related-HCV	3165	3010–3141	6973	6715–6938	11,444	11,014–11,393	97	94	86
*YLLs*									
Cirrhosis	588	304–569	665	368–656	394	202–398	44	38	33
HCC	429	177–410	576	308–560	326	158–334	40	40	41
Transplant	7	0–0	47	0–35	51	0–36	14	6	6
Total HCV related	1024	613–996	1288	848–1280	771	504–778	42	38	35
Total not related-HCV	12,231	10,654–12,191	9822	8567–9840	8730	7553–8716	97	90	80
*YLDs*									
HCV+ acute	198	181–198	97	84–97	75	61–75	100	100	100
HCV+ chronic	1682	1541–1675	1664	1497–1664	1139	975–1142	52	43	40
Cirrhosis	250	205–246	423	350–421	428	328–433	41	35	30
HCC	13	7–13	21	12–20	17	10–18	38	38	41
Transplant	1	0–1	6	1–4	8	3–7	0	0	0
Total HCV related	2144	1970–2135	2210	1982–2210	1667	1440–1672	55	44	40
*DALYs*									
HCV+ acute	198	181–198	97	84–97	75	61–75	100	100	100
HCV+ chronic	1682	1541–1675	1664	1497–1664	1139	975–1142	52	43	40
Cirrhosis	838	540–815	1088	761–1074	822	572–832	43	37	32
HCC	442	184–423	597	325–580	343	173–352	40	40	41
Transplant	8	0–1	53	1–39	59	3–42	12	6	5
Total HCV related	3168	2674–3128	3498	2950–3496	2438	2038–2452	51	41	38

*HCV: hepatitis C infection, PWID: people who inject drugs, HCC: hepatocellular carcinoma; YLDs: years lost of disabilities; YLLs: years of life lost; DALYs; disability adjusted life years* ^a^: Proportion that is attributable to active PWIDs

**Fig. 5:**
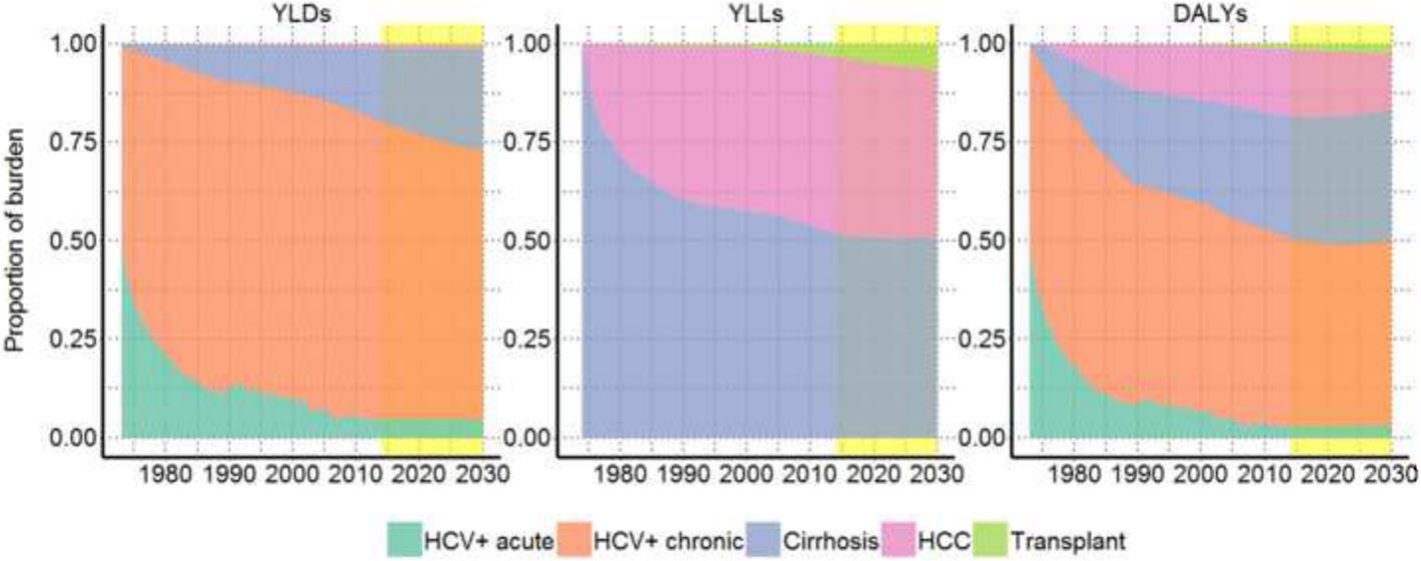
Proportion of burden attributable to health states among people who inject drugs in Norway, 1973–2030. *Hepatitis C: HCV, PWID: people who inject drugs. YLDs: years lived with disabilities; YLLs: years of life lost; DALYs; disability adjusted life years. Health states: acute HCV in dark green, chronic HCV in orange, cirrhosis in purple, hepatocellular carcinoma in pink, received a liver transplants in light green*. *Yellow shaded area shows predictions*

Our estimates show that chronic HCV infection contributes most to the total burden of HCV infection peaking with 1917 DALYs (51.8%) in 2007 and falling to 1139 DALYs (46.7%) in 2030 (Fig. 5). Cirrhosis was estimated to peak with 1109 DALYs (30.1%) in 2011 and fall to 822 DALYs (33.7%) in 2030. HCC is estimated to peak with 610 DALYs (16.6%) in 2011 and fall to 343 DALYs (14.1%) in 2030. Chronic HCV infection contributed most to the YLDs, while death due to HCV related cirrhosis accounts for most of the YLLs (Fig. 5).

**Fig. 6:**
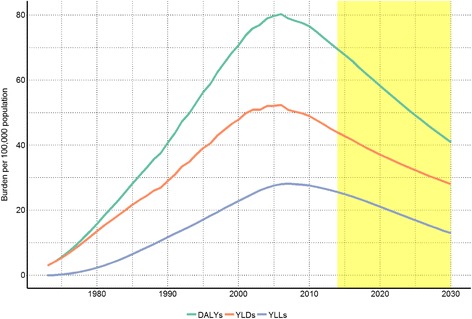
Burden of hepatitis C associated with injecting drug use per 100,000 population in Norway, 1973–2030. *YLDs: years lived with disabilities in orange; YLLs: years of life lost in purple; DALYs; disability adjusted life years in green*. *Yellow shaded area shows predictions, using future population estimates calculated by Statistics Norway*

We used our estimates of total DALYs, YLLs and YLDs among PWIDs to calculate the burden of HCV caused by injecting drug use in Norway (Fig. 6). The total burden increased since 1973 and peaked in 2006 with 80.36 DALYS per 100,000 inhabitants. Since then the burden of HCV related to injecting drug use has been decreasing, but is still estimated to be 71.29 DALYs per 100,000 in 2013. We provide detailed estimates for all years in csv files in the supplemental material (Additional files 3, 4, 5, 6, 7, 8, 9), and all code and results is available at https://github.com/raubreywhite/hcv_burden_paper_2017.

## Discussion

The estimated total HCV burden related to injecting drug use was 67.71 DALYs per 100,000 inhabitants in Norway in 2015. Chronic HCV infection contributed most to the total burden of HCV infection (47%) and an estimated 381 PWIDs (4.5% of all PWIDs and 7.6% of susceptible PWIDs per year) are newly infected with HCV annually.

The Global Burden of Diseases, Injuries, and Risk Factors Study (GBD) [22, 23] estimated that ‘Communicable, maternal, neonatal and nutritional disorders’ burden was 1055 DALYs per 100,000 in Norway (4% of all DALYs) in 2010. We estimated that the HCV burden related to injecting drug use was 76.61 DALYs per 100,000 in the same year, illustrating the impact of HCV infection in a relatively small risk group on the total population. The estimated burden of acute HCV infection was 2.31 DALYs per 100,000 by GBD versus our estimate of 2.70 DALYs per 100,000. The GBD estimates that cirrhosis and HCC due to HCV results in 53.97 and 22.51 DALYs per 100,000 respectively, compared with our estimates of 22.81 DALYs per 100,000 for cirrhosis and 12.47 DALYs per 100,000 for HCC in Norway. Even though our estimates diverged from the GBD study, they were in the same order of magnitude (i.e. both between 10 and 100 DALYs per 100,000); the total burden estimated by GBD was 79 compared to our estimated 38 DALYs per 100,000 for the same parameters, which supports the validity of our results. Another study estimated that burden of HCV in Norway in 2004 was 40.25 DALYs per 100,000, but only included acute HCV infection, cirrhosis and HCC and not chronic HCV infection [24]. When combining these sequela, our estimate would be 79.06 DALYs per 100,000 in 2004, suggesting that estimates from our model are in line with previous studies using different methods.

The Norwegian surveillance system of HCV cannot provide an accurate picture of the current incidence and prevalence of HCV due to asymptomatic nature of the disease. This model relied on outcomes more likely to be diagnosed, such as cirrhosis and HCC, to estimate the incidence and prevalence of HCV infection. Our model provided estimates on the number of newly infected PWIDs and the total number of PWIDs with HCV. Those figures could be compared with the numbers reported to the Norwegian surveillance system and used to estimate the underreporting or underdiagnoses of HCV in Norway.

In our model, people who clear HCV or have a successful treatment return to the HCV negative susceptible group. Results from studies of reinfection risk after spontaneous clearance in PWIDs are conflicting [19]. We choose to consider the risk of reinfection the same as risk of infection, as we had no observable data on reinfection to fit against, we preferred to be on the side of caution. Furthermore, due to low treatment numbers, we did not expect this to have a material impact on our estimates.

We used data on HCV associated cirrhosis among PWIDs from one hospital whose catchment area has more immigrants from high incidence countries compared to the rest of Norway. While we included only those with a history of injecting drug use, these numbers may still over-represent immigrants from high incidence countries compared to data from other Norwegian hospitals. As such, care should be taken when interpreting our results. However, no transition probabilities relating to cirrhosis were estimated to be on the boundaries as defined by our literature search. Therefore, we have confidence that our estimates are not biased to the point that they are impossible according to the literature.

We used data on LTX in Norway from NLTR to fit our model [25]. The number of individuals receiving LTX is not only related to demand, but also to the availability. We therefore included an increased transition rate for receiving a LTX as availability increased over time. Active PWIDs are not eligible for LTX and 6 months of abstinence (with some exceptions) is required for those with alcohol abuse in Norway. This could result in many HCV positive cirrhosis or HCC patients not being eligible for LTX. In addition, we only took into account mortality in the first year after the LTX, whereas mortality in the years following LTX is also increased compared to the general population, especially among those with HCV infection due to re-infection of the graft [20]. Therefore, our mortality estimates due to LTX are likely to be underestimated [20].

One limitation of our model is that data came from a number of different sources, each with their own biases, delays in registration, and underreporting. Our model treated each data source as equally trustworthy and did not correct for any underreporting, as we did not have accurate numbers for underreporting in the various compartments. Experts in the field estimate that 50% of all cirrhosis cases are not reported (personal communication). In that case, our model may have underestimated the actual HCV burden associated with injecting drugs in Norway. Undiagnosed or unreported cirrhosis and HCC is thus an important limiting factor in estimating the actual burden and should therefore be further studied. Data sources used to estimate HCC, HCC death, and cirrhosis death did not report HCV specific numbers. We therefore used a correction factor based on literature [25, 26]. Furthermore, some of our data sources provided prevalence data. Fitting natural history models to prevalence data is known to create artificially small confidence intervals. Caution should therefore be used when making inferences based on the confidence intervals.

Most information from Norway is based on studies from Oslo. Oslo used to account for approximately 50% of all PWIDs in Norway in the mid-90s. This pattern changed over the recent years to an estimated 20% in 2013 (unpublished data) [27]. We added how disperse the spread of PWIDs is for each year based on data from the drug related deaths. The HCV surveillance data from MSIS indicates that PWIDs in Oslo are older than those reported in other parts of the country (unpublished MSIS data). This could have affected our estimates on HCV disease burden in Norway. Differences in age distribution could also have resulted in an underestimation of the burden.

Our model forecasted the situation up to 2030, with current treatment and control measures, including harm reduction, testing and follow-up. Changes in these strategies may influence the burden estimates. Changes in behaviour, risk population or the type of drugs used can also affect the impact of HCV in the future. With these substantial limitations in mind, and keeping the well-known issues with extrapolation in mind, the extrapolated results out to 2030 may be of interest due to the slow progression of HCV disease and subsequent sequelae.

## Conclusion

We have constructed a model to better estimate the burden of HCV infections among PWIDs in Norway. This model can be used to estimate the impact and cost-benefit of various interventions on the burden of HCV in Norway in order to assist public health decision. Especially important would be estimating the impact of the new DAA’s treatment regimens on the HCV burden in Norway, upscaling treatment, treating everyone or only subgroups, such as only those with advance liver disease.

## Additional files

Additional file 1: Figure legends for supplementary figures. (S1-S5). **Figure S1.** Needle and syringe exchange programmes coverage and GINI coefficient of drug deaths in Norway, 1973–2030. **Figure S2.** Fitting estimated number of people who inject drugs with reported numbers in Norway, 1973–2030. **Figure S3.** Mean age of injecting debut among people who inject drugs in Norway, 1973–2013. **Figure S4.** Sensitivity analyses. **Figure S5.** Mean estimated age of people who inject drugs in Norway, 1973–2030. (ZIP 125 kb)

Additional file 2: **Method S1.** Detailed description of the methods used in this manuscript. (DOCX 25 kb)

Additional file 3: **Table S1.** Summary of model estimates from the compartmental model to estimate the burden of hepatitis C associated with people who inject drugs in Norway. (DOCX 21 kb)

Additional file 4: Summary of all estimates. Summary of all estimates. (CSV 50 kb)

Additional file 5: Machine friendly summary of all estimates. Machine friendly summary of all estimates. (CSV 444 kb)

Additional file 6: Summary of lower estimates. (CSV 64 kb)

Additional file 7: Machine friendly summary of lower estimates. Machine friendly summary of lower estimates. (CSV 443 kb)

Additional file 8: Summary of upper estimates. (CSV 65 kb)

Additional file 9: Machine friendly summary of upper estimates. (CSV 446 kb)

## Abbreviations

CI: Confidence interval
DAAs: Direct-acting antivirals
DALYs: Disability adjusted life years
GBD: The Global Burden of Diseases, Injuries, and Risk Factors Study
HCC: Hepatocellular carcinoma
HCV: Hepatitis C virus
LTX: Liver transplantation
MSIS: Norwegian Surveillance System for Communicable Diseases
NCR: Norwegian Cancer Registry
NLTR: Nordic Liver Transplant Registry
NPR: National Patient Registry
PWIDs: People who inject drugs
YLDs: Years lived with disabilities
YLLs: Years of life lost

## References

[1] Polaris Observatory HCVC (2017). Global prevalence and genotype distribution of hepatitis C virus infection in 2015: a modelling study. Lancet Gastroenterol Hepatol.

[2] Lozano R, Naghavi M, Foreman K, Lim S, Shibuya K, Aboyans V, Abraham J, Adair T, Aggarwal R, Ahn SY (2012). Global and regional mortality from 235 causes of death for 20 age groups in 1990 and 2010: a systematic analysis for the global burden of disease study 2010. Lancet.

[3] World Health Organization: Factsheet: Hepatitis C. 2016. http://www.who.int/mediacentre/factsheets/fs164/en/. Accessed 31 May 2017.

[4] Seeff LB (2002). Natural history of chronic hepatitis C. Hepatology.

[5] Thein HH, Yi Q, Dore GJ, Krahn MD (2008). Estimation of stage-specific fibrosis progression rates in chronic hepatitis C virus infection: a meta-analysis and meta-regression. Hepatology.

[6] Fattovich G, Giustina G, Degos F, Tremolada F, Diodati G, Almasio P, Nevens F, Solinas A, Mura D, Brouwer JT (1997). Morbidity and mortality in compensated cirrhosis type C: a retrospective follow-up study of 384 patients. Gastroenterology.

[7] Hu KQ, Tong MJ (1999). The long-term outcomes of patients with compensated hepatitis C virus-related cirrhosis and history of parenteral exposure in the United States. Hepatology.

[8] Brown RS (2005). Hepatitis C and liver transplantation. Nature.

[9] Nelson PK, Mathers BM, Cowie B, Hagan H, Des Jarlais D, Horyniak D, Degenhardt L (2011). Global epidemiology of hepatitis B and hepatitis C in people who inject drugs: results of systematic reviews. Lancet.

[10] Shepard CW, Finelli L, Alter MJ (2005). Global epidemiology of hepatitis C virus infection. Lancet Infect Dis.

[11] Dalgard O, Jeansson S, Skaug K, Raknerud N, Bell H (2003). Hepatitis C in the general adult population of Oslo: prevalence and clinical spectrum. Scand J Gastroenterol.

[12] Hans B, Hilde K, Venelina K, Øivind N, Synne S, Kathrine S-J, Martin S, Kristian SP, VIS S, Folkehelseinstitutt N (2013). Årsrapport 2012 for sykdomsprogrammet: Blod- og seksuelt overførbare infeksjoner.

[13] Martin NK, Vickerman P, Dore GJ, Grebely J, Miners A, Cairns J, Foster GR, Hutchinson SJ, Goldberg DJ, Martin TC (2016). Prioritization of HCV treatment in the direct-acting antiviral era: an economic evaluation. J Hepatol.

[14] Rein DB, Wittenborn JS, Smith BD, Liffmann DK, Ward JW (2015). The cost-effectiveness, health benefits, and financial costs of new antiviral treatments for hepatitis C virus. Clin Infect Dis.

[15] Najafzadeh M, Andersson K, Shrank WH, Krumme AA, Matlin OS, Brennan T, Avorn J, Choudhry NK (2015). Cost-effectiveness of novel regimens for the treatment of hepatitis C virus. Ann Intern Med.

[16] Deuffic-Burban S, Obach D, Canva V, Pol S, Roudot-Thoraval F, Dhumeaux D, Mathurin P, Yazdanpanah Y. Cost-effectiveness and budget impact of interferon-free direct-acting antiviral-based regimens for hepatitis C treatment: the French case. J Viral Hepat. 2016; 10.1111/jvh.12546 27144512

[17] Engen ØB: [gets 50 percent rebate on hepatitis C medication] in Norwegian. In: Dagens Medisin*.* 2016.

[18] Zhu C, Richard B, Lu P, Jorge N (1997). Algorithm 778: L-BFGS-B: Fortran subroutines for large-scale bound-constrained optimization. ACM Trans Math Softw.

[19] Grebely J, Prins M, Hellard M, Cox AL, Osburn WO, Lauer G, Page K, Lloyd AR, Dore GJ, International collaboration of incident HIV (2012). hepatitis C virus clearance, reinfection, and persistence, with insights from studies of injecting drug users: towards a vaccine. Lancet Infect Dis.

[20] Aberg F, Gissler M, Karlsen TH, Ericzon BG, Foss A, Rasmussen A, Bennet W, Olausson M, Line PD, Nordin A (2015). Differences in long-term survival among liver transplant recipients and the general population: a population-based Nordic study. Hepatology.

[21] Fosby B, Melum E, Bjoro K, Bennet W, Rasmussen A, Andersen IM, Castedal M, Olausson M, Wibeck C, Gotlieb M (2015). Liver transplantation in the Nordic countries - an intention to treat and post-transplant analysis from the Nordic liver transplant registry 1982-2013. Scand J Gastroenterol.

[22] Evaluation. IfHMa: Global Burden of Disease (GBD). 2016. Access: 01.06.2016.

[23] Salomon JA, Vos T, Hogan DR, Gagnon M, Naghavi M, Mokdad A, Begum N, Shah R, Karyana M, Kosen S (2012). Common values in assessing health outcomes from disease and injury: disability weights measurement study for the global burden of disease study 2010. Lancet.

[24] Muhlberger N, Schwarzer R, Lettmeier B, Sroczynski G, Zeuzem S, Siebert U (2009). HCV-related burden of disease in Europe: a systematic assessment of incidence, prevalence, morbidity, and mortality. BMC Public Health.

[25] Eskesen AN, Bjoro K, Aandahl EM, Line PD, Melum E (2014). Low use of surveillance and early diagnosis of hepatocellular carcinoma in Norway--a population-based cohort study. Cancer Epidemiol.

[26] Haukeland JW, Lorgen I, Schreiner LT, Frigstad SO, Brandsaeter B, Bjoro K, Bang C, Raknerud N, Konopski Z (2007). Incidence rates and causes of cirrhosis in a Norwegian population. Scand J Gastroenterol.

[27] Amundsen EJ, Bretteville-Jensen AL (2010). Hard drug use in Norway. NAT.

[28] Gjersing L, Bretteville-Jensen AL (2014). Gender differences in mortality and risk factors in a 13-year cohort study of street-recruited injecting drug users. BMC Public Health.

[29] Martin NK, Vickerman P, Miners A, Foster GR, Hutchinson SJ, Goldberg DJ, Hickman M (2012). Cost-effectiveness of hepatitis C virus antiviral treatment for injection drug user populations. Hepatology.

[30] Seeff LB (2009). The history of the "natural history" of hepatitis C (1968-2009). Liver Int.

[31] Kielland KB, Amundsen EJ, Dalgard O (2014). HCV treatment uptake in people who have injected drugs - observations in a large cohort that received addiction treatment 1970-1984. Scand J Gastroenterol.

[32] Hutchinson SJ, Bird SM, Goldberg DJ (2005). Modeling the current and future disease burden of hepatitis C among injection drug users in Scotland. Hepatology.

[33] Sangiovanni A, Prati GM, Fasani P, Ronchi G, Romeo R, Manini M, Del Ninno E, Morabito A, Colombo M (2006). The natural history of compensated cirrhosis due to hepatitis C virus: a 17-year cohort study of 214 patients. Hepatology.

