# Supplementary figures and images for: Modelling the burden of hepatitis C infection among people who inject drugs in Norway, 1973–2030
Source: BMC Infect Dis. 2017 Aug 3;17:541. doi: 10.1186/s12879-017-2631-2 (PMC5543437; doi:10.1186/s12879-017-2631-2)

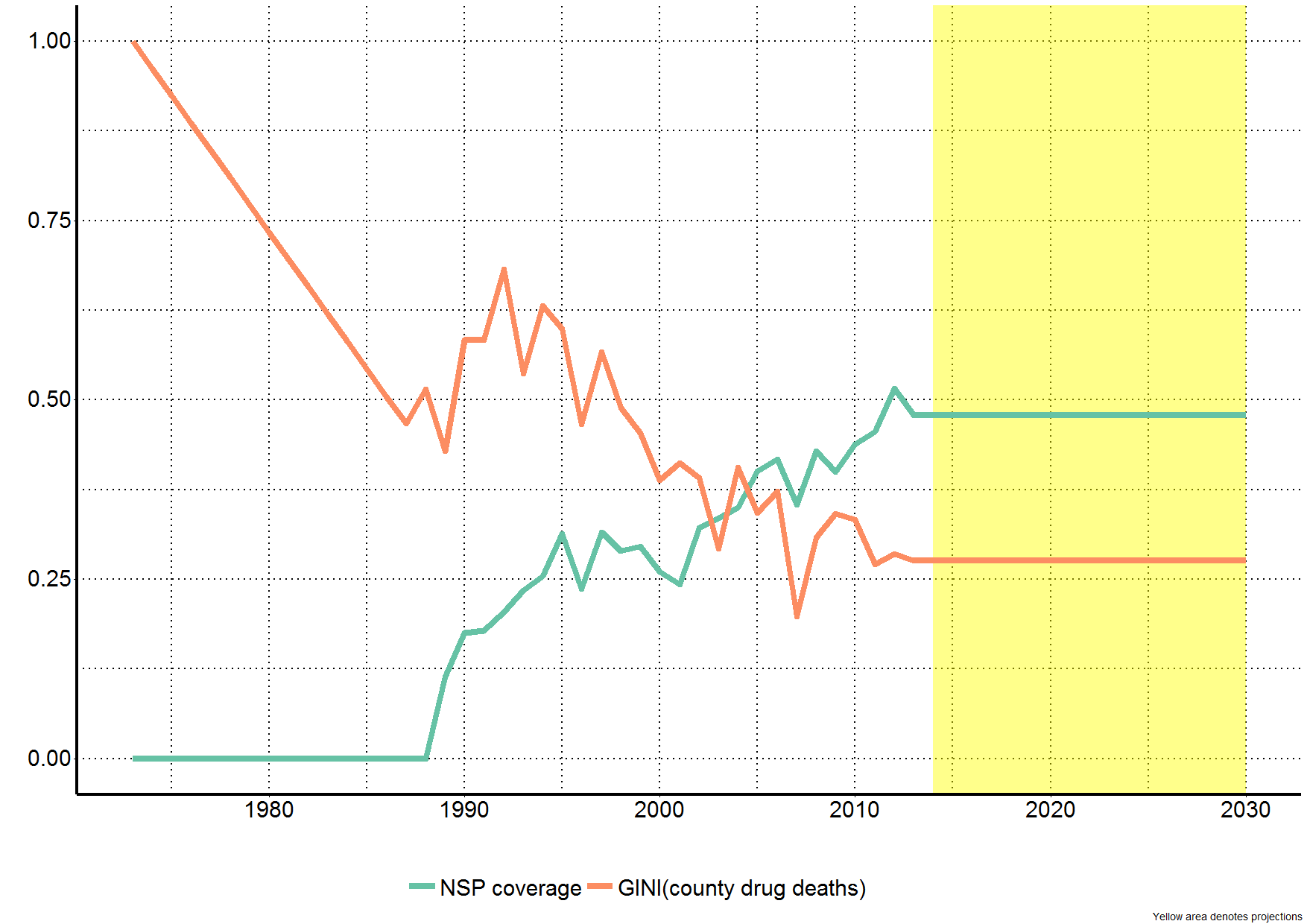

Supplement: Supplementary file 1 — Figure legends for supplementary figures. (S1-S5). Figure S1. Needle and syringe exchange programmes coverage and GINI coefficient of drug deaths in Norway, 1973–2030. Figure S2. Fitting estimated number of people who inject drugs with reported numbers in Norway, 1973–2030. Figure S3. Mean age of injecting debut among people who inject drugs in Norway, 1973–2013. Figure S4. Sensitivity analyses. Figure S5. Mean estimated age of people who inject drugs in Norway, 1973–2030. (ZIP 125 kb) [file 12879_2017_2631_MOESM1_ESM.zip › Figure S1R2.png]

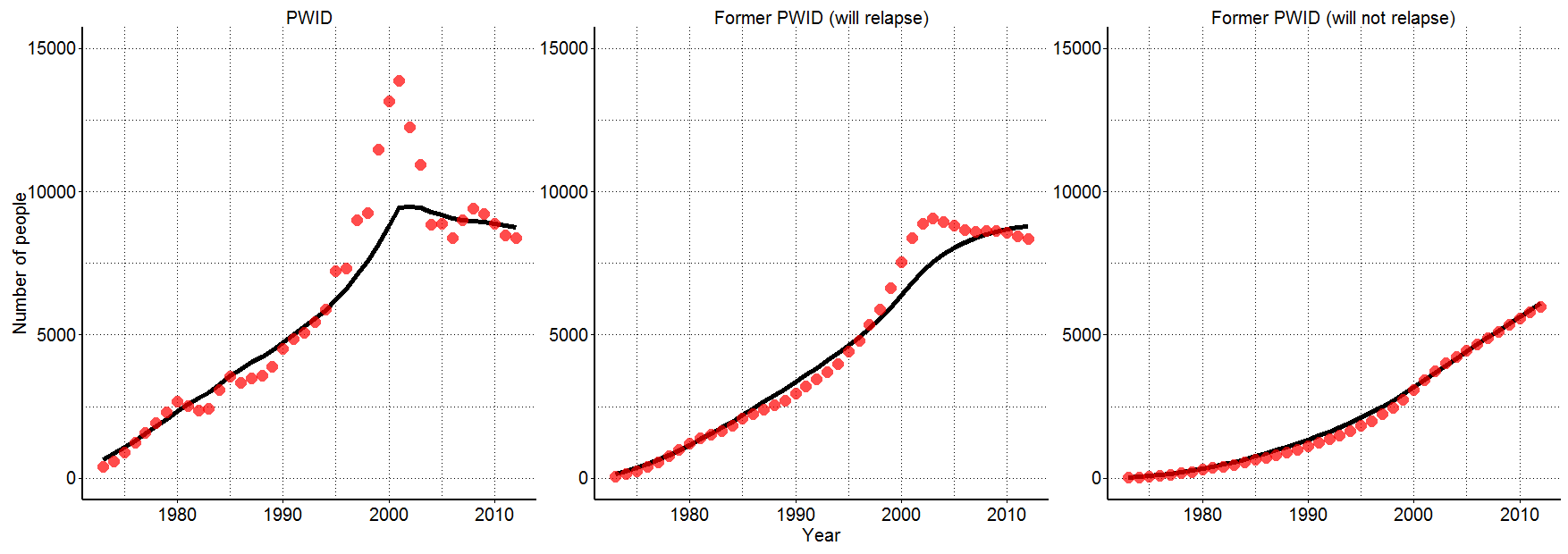

Supplement: Supplementary file 1 — Figure legends for supplementary figures. (S1-S5). Figure S1. Needle and syringe exchange programmes coverage and GINI coefficient of drug deaths in Norway, 1973–2030. Figure S2. Fitting estimated number of people who inject drugs with reported numbers in Norway, 1973–2030. Figure S3. Mean age of injecting debut among people who inject drugs in Norway, 1973–2013. Figure S4. Sensitivity analyses. Figure S5. Mean estimated age of people who inject drugs in Norway, 1973–2030. (ZIP 125 kb) [file 12879_2017_2631_MOESM1_ESM.zip › Figure s2R2.png]

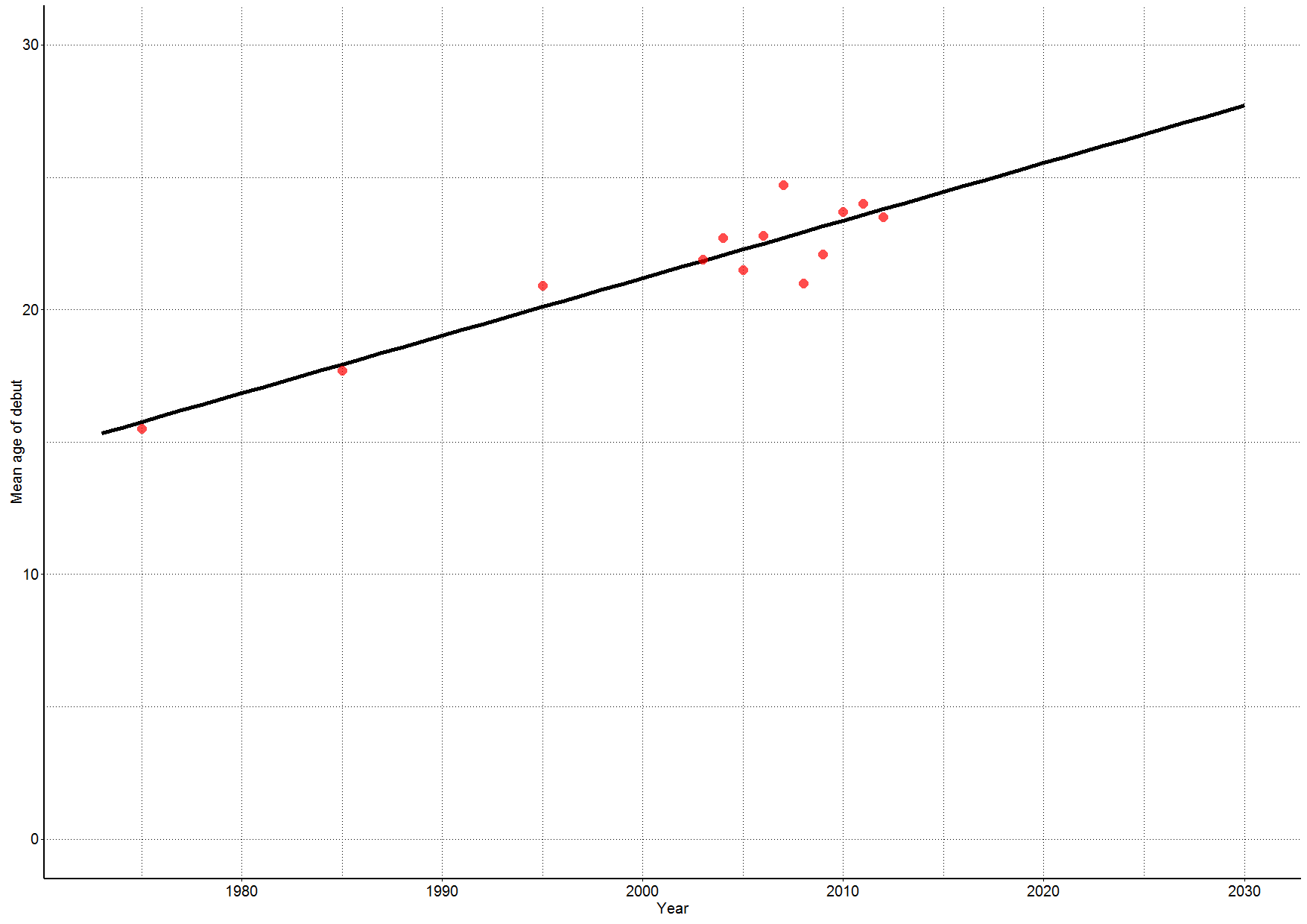

Supplement: Supplementary file 1 — Figure legends for supplementary figures. (S1-S5). Figure S1. Needle and syringe exchange programmes coverage and GINI coefficient of drug deaths in Norway, 1973–2030. Figure S2. Fitting estimated number of people who inject drugs with reported numbers in Norway, 1973–2030. Figure S3. Mean age of injecting debut among people who inject drugs in Norway, 1973–2013. Figure S4. Sensitivity analyses. Figure S5. Mean estimated age of people who inject drugs in Norway, 1973–2030. (ZIP 125 kb) [file 12879_2017_2631_MOESM1_ESM.zip › Figure s3R2.png]

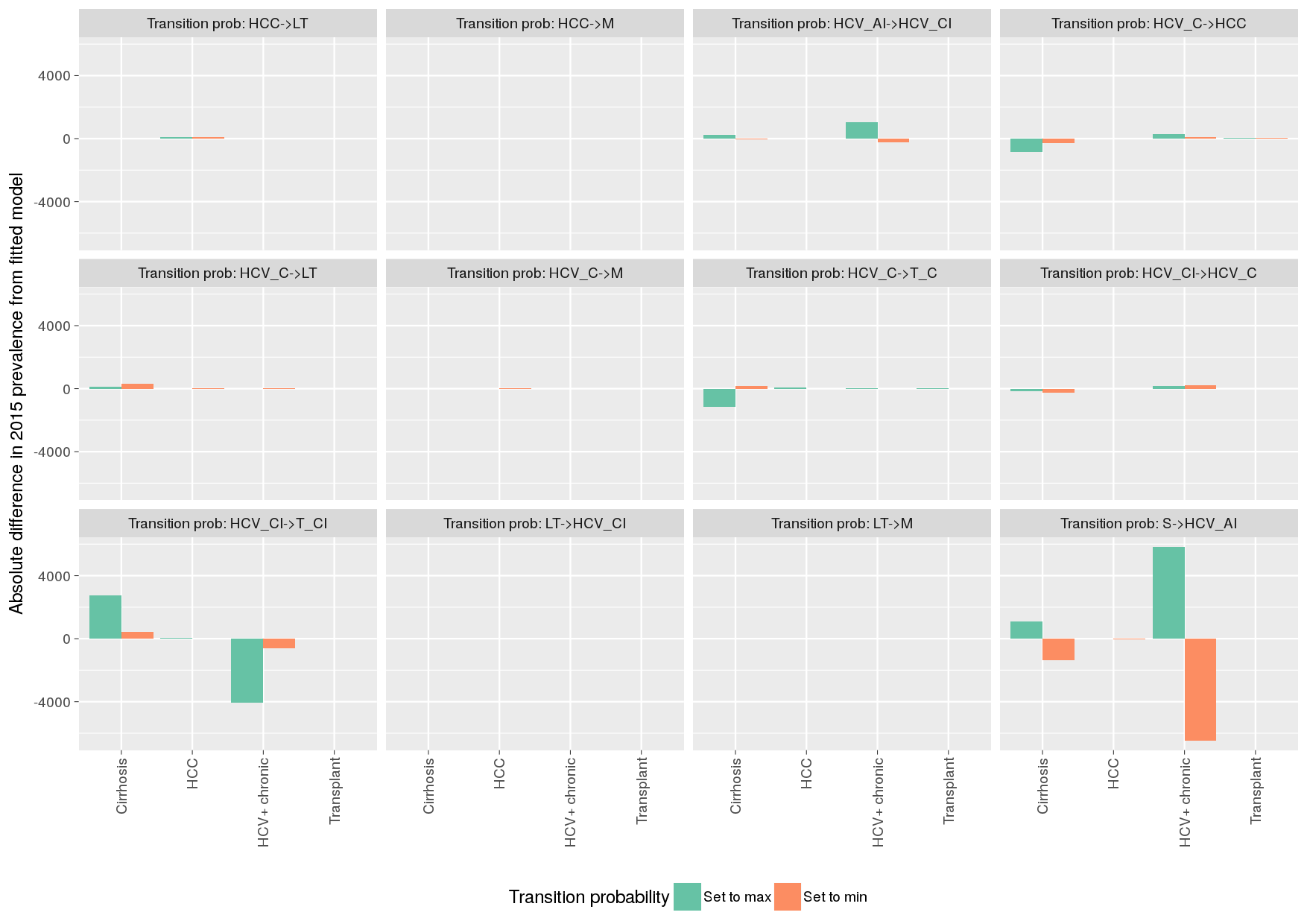

Supplement: Supplementary file 1 — Figure legends for supplementary figures. (S1-S5). Figure S1. Needle and syringe exchange programmes coverage and GINI coefficient of drug deaths in Norway, 1973–2030. Figure S2. Fitting estimated number of people who inject drugs with reported numbers in Norway, 1973–2030. Figure S3. Mean age of injecting debut among people who inject drugs in Norway, 1973–2013. Figure S4. Sensitivity analyses. Figure S5. Mean estimated age of people who inject drugs in Norway, 1973–2030. (ZIP 125 kb) [file 12879_2017_2631_MOESM1_ESM.zip › Figure s4R2.png]

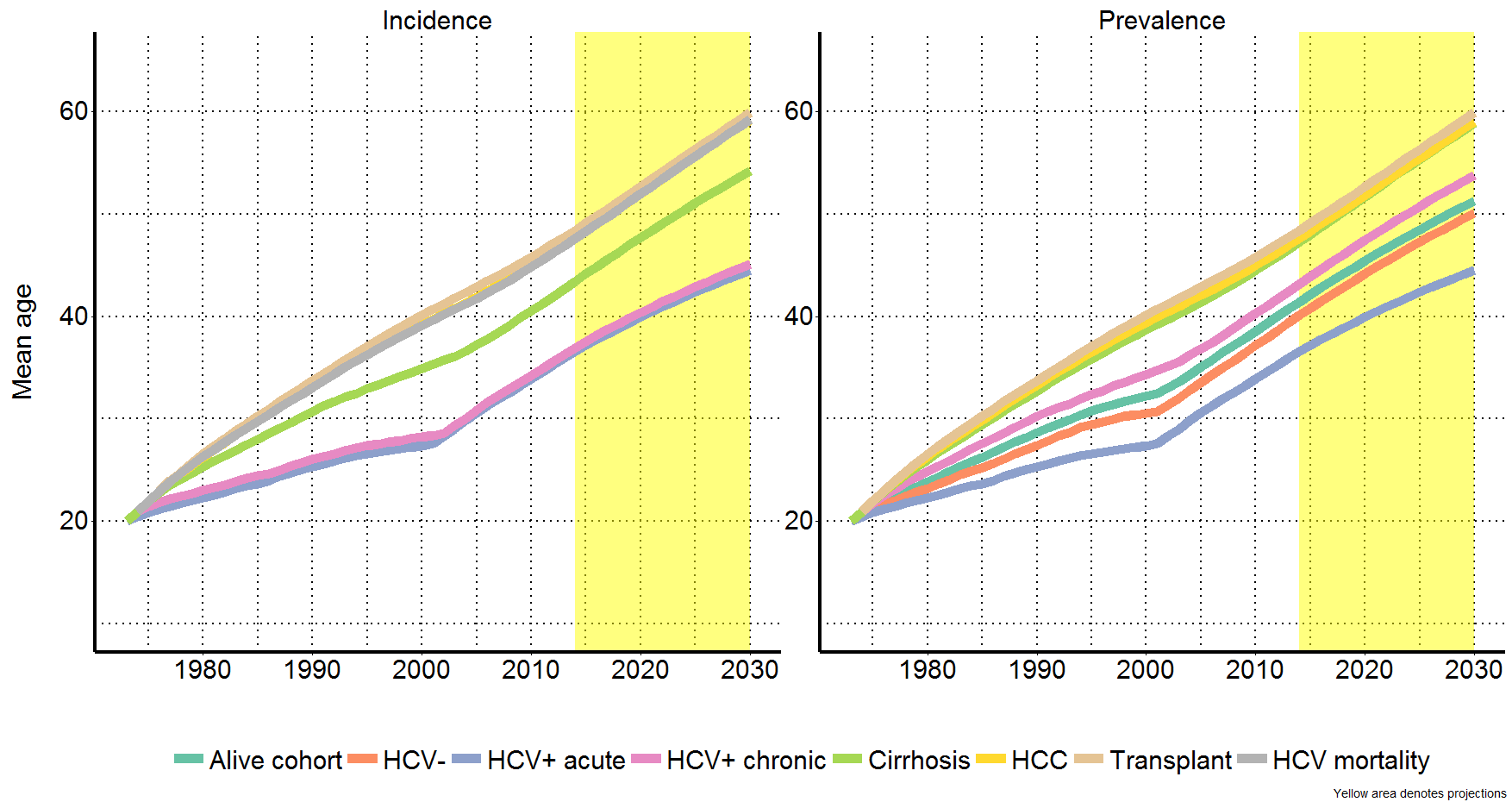

Supplement: Supplementary file 1 — Figure legends for supplementary figures. (S1-S5). Figure S1. Needle and syringe exchange programmes coverage and GINI coefficient of drug deaths in Norway, 1973–2030. Figure S2. Fitting estimated number of people who inject drugs with reported numbers in Norway, 1973–2030. Figure S3. Mean age of injecting debut among people who inject drugs in Norway, 1973–2013. Figure S4. Sensitivity analyses. Figure S5. Mean estimated age of people who inject drugs in Norway, 1973–2030. (ZIP 125 kb) [file 12879_2017_2631_MOESM1_ESM.zip › Figure s5R2.png]
