# Supplementary material for: Modelling the burden of hepatitis C infection among people who inject drugs in Norway, 1973–2030
Source: BMC Infect Dis. 2017 Aug 3;17:541. doi: 10.1186/s12879-017-2631-2 (PMC5543437; doi:10.1186/s12879-017-2631-2)
Supplement: Supplementary file 2 — Method S1. Detailed description of the methods used in this manuscript. (DOCX 25 kb) [file 12879_2017_2631_MOESM2_ESM.docx]

### Supplemental Methods

#### Markov model

We constructed a Markov model for the natural history model of HCV, which follows the disease dynamics in a population over time, in former and current PWID including compartments with eight different HCV-related health states (HCV negative susceptible, acute HCV infection, chronic HCV infection, HCV positive and cirrhosis, HCV negative and cirrhosis, HCC, liver transplant, HCV related mortality; figure 1). The annual transition probabilities between compartments were estimated using a numerical optimizer [11] (details further below) where the probability estimates were restricted to be within bounds determined by previous estimates in the literature (Table 2).

The first compartment contains HCV negative, susceptible, current PWIDs, which has an influx of newly started PWIDs (Figure 1). Among active PWIDs, there is a risk of contracting HCV and transfer to the compartment with acute HCV infection. These people will either clear infection and return to the HCV negative (susceptible) group or develop a chronic HCV infection. People are classified as having chronic infection if the HCV infection is not spontaneously cleared within one year and if they have not developed cirrhosis. As we are focused on HCV infections associated with drug use, we assume that former PWIDs have no risk of acquiring HCV infection, unless they resume injecting drugs and return to the active PWIDs part of the model. Individuals with chronic infection can develop cirrhosis and subsequently HCC. Former PWIDs with cirrhosis and/or HCC can receive treatment for the liver disease by getting a liver transplant (LTX). Individuals can die from HCV related causes after developing cirrhosis, HCC or after LTX. Individuals receiving a liver transplant have an increased risk of mortality for the following year, mainly related to surgical and early post-operative complications. HCV related mortality in individuals with cirrhosis is reduced by a factor of five after successfully receiving HCV treatment and becoming HCV negative [12].

From chronic HCV and chronic HCV with cirrhosis, individuals can receive successful HCV treatment and return to the respective HCV negative, susceptible compartment. Age-specific mortality is taken into account, including additional risk of mortality for PWIDs. The model is replicated for active PWIDs, former PWIDs who will relapse, and former PWIDs who will never relapse. In all stages of the disease individuals can either stop injecting drugs (i.e. transfer from active PWIDs to former PWIDs) or start injecting drugs (i.e. transfer from former PWIDs who will relapse to active PWIDs) or stop with injecting completely (i.e. transfer from PWIDs to former PWIDs who will never relapse). In addition, the model considers HCV infection with three different genotypes that have distinct HCV treatment probabilities, successful or unsuccessful treatment and the model is age-stratified into one-year age groups (1-100 years).

#### Data sources and parameter estimation

###### Drug related deaths and infectivity of PWIDs

The probability of someone transitioning from susceptible to infected was calculated through the following algorithm:

p_new1_=1−(1−p_old_)^10×(Number PWID With HCV/ Number PWID) ×GINI Deaths Drugs By County^

p_old_ is the probability that a person is infected by one PWID (displayed in Table 2). We assume that a PWID has a social circle of 10 other PWIDs. We therefore adjust this probability to be the probability that a person is infected by at least one of ten PWIDs (the number 10 is arbitrary and p_old_ will scale to whatever number we choose). We then calculate the Gini coefficient of county-level drug deaths for each year and used this to model the infectivity. We used the annual number of drug-related deaths in each Norwegian county as a proxy for the size of the injecting epidemic (e.g. number of PWIDs) in those regions.[29] If all drug deaths occurred in one county, then the Gini coefficient would be one, and the infectivity high. As drug deaths become more equally distributed throughout the country, the Gini coefficient drops, and so does infectivity.

The Gini coefficient variable was included to damper the homogeneous mixing assumption without adding too much complexity to the model. Without the Gini coefficient variable the percentage of PWIDs with HCV was consistently overestimated by 15-20 percentage points. The time series for the Gini coefficient is displayed in Figure S1.

###### Drug related deaths and needle and syringe program coverage

We used the annual number of drug-related deaths in each Norwegian county as a proxy for the size of the injecting epidemic in each county. As of 2013, the four areas in Norway with the most PWIDs (Oslo, Bergen, Trondheim, and Stavanger/Sandnes) are running needle and syringe exchange programs with complete coverage of the PWIDs in their catchment areas. That is, free needles and syringes are available for all PWIDs who want them. For Oslo, we had approximate numbers of needles handed out every year, so we constructed a new variable: annual needles handed out per annual drug related deaths. Assuming full coverage in 2013, we extrapolated the 2013 association between annual needles handed out per annual drug related deaths and 100% NSP coverage into historical NSP coverage proportions. For Bergen, Trondheim, and Stavanger/Sandnes, we did not have historical coverage proportions, so we assumed that coverage started in 1992 (at zero) and increased in a linear manner to full coverage in 2013.

Taking into account time-series of needle exchange program coverage in the four major areas (assuming no coverage in other areas of Norway) and an estimation of the proportion of the total PWIDs in each of these regions, we estimated the needle exchange coverage for all of Norway (Figure S1). This variable was included as a modifying factor against infectiousness, and its efficacy was estimated from the data. [29,30] The infectiousness transition probability was modified in the following manner:

p_new2_=p_new1_×(1- Estimated Coefficient × Program Coverage)

###### People who inject drugs

Norwegian Institute for Alcohol and Drug Research (SIRUS) has estimated the number of new PWIDs and prevalence of active PWIDs, former PWIDs who will relapse, and former PWIDs who will never relapse for each year from 1973 to 2013. These estimates were not internally consistent, so we estimated transition rates to best fit the non-consistent data, and in-turn obtain internally consistent estimates for the PWID envelope (Figure S2). We extended and repeated the number of new PWIDs in 2013 through to 2030.

###### Age of injecting debut

Age of injecting debut was taken from SIRUS estimates in 1975, 1985, and 1995, and the life quality report from 2003 to 2012. A relatively linear trend in the age of debut was observed over time, so we fit a linear regression model to the data and predicted mean age of injecting debut from 1973 to 2030 (Figure S3).

###### PWIDs with HCV

The proportion of PWIDs with HCV RNA was based on data collected through cross sectional health studies among PWIDs attending low threshold harm reduction-based health care centers in Oslo targeted towards drug users [10].

###### Cirrhosis

We received data from one hospital (Akershus universitetssykehus HF) on the number of PWIDs (current and former) treated with cirrhosis associated with HCV in 2013. From this number we estimated the total number of cases treated in Norway that year to be ten time larger as the hospital has a catchment area of 10% of the Norwegian population.

###### HCC disease

Aggregated data on the number of people with liver cancer or HCC were obtained from the Norwegian cancer registry (NCR) using ICD10 code C22 (Malignant neoplasm of liver and intrahepatic bile ducts, which includes HCC). Numbers were adjusted according to the proportion of HCC among those with ICD10 code C22 (77%) and for disease attributable to HCV in Norway (26%), resulting in 20% of the extracted data estimated to be HCV associated HCC [21].

###### Liver transplants

All liver transplants (LTX) in Norway are performed in one hospital (Oslo University Hospital, Rikshospitalet). Data on all patients receiving a liver allograft since the start of the program in 1984 and patients entering the waiting list after 1990 have been recorded. In Nordic Liver Transplant Registry (NLTR).[20] We used aggregated data on all liver transplants performed from 2000 to 2013 in Norway, including information on listing diagnosis (HCV cirrhosis and HCC). We used the number of patients with antibodies against HCV as targets for liver transplants in the model. Mortality within the first year after the LTX is considered as HCV related mortality. After the first year the individuals who received a LTX have the same risk as the rest of the population in the model.

###### Cirrhosis mortality

Aggregate data on death entries (2000-2013) were obtained from the “Norwegian Cause of Death Registry”. We included individuals with an underlying cause of cirrhosis (ICD10 codes K74.3, K74.4, K74.5 and K74.6). The mortality numbers were then adjusted to the attributable risk of hepatitis C (14%) and used as a target in the model [18].

###### HCC mortality

Aggregate data on death entries (2000-2013) were obtained from the “Cause of Death Registry”. We included individuals with an underlying cause of HCC (ICD10 code C22). The mortality numbers were adjusted for HCC among those with ICD10 code C22 (77%) and for to the attributable risk of hepatitis C (26%), before we used them as a target in the model [21].

###### Treatment rates

The Norwegian prescription database (NorPD) was used to estimate the treatment rates for HCV in Norway. Genotype distribution of HCV was based on data from the NIPH.[10] We estimated treatment success for each year, combining the absolute number of treatments from NorPD with treatment response and duration of treatment per genotype.

#### Disability weightings

Each health stage in the model can be assigned with a disability weight to calculate the disability adjusted life years (DALYs), which provides a quantitative measure of health loss due to disease. DALYs estimate years of life lost (YLLs) due to premature death, as well as years of healthy life lost due to disability from disease and injury (years lived with disabilities: YLDs). The model output can be used to assess and compare the relative impact of different diseases, injuries and risk factors on populations. Using the Global Burden of Disease study 2010 disability weights [13], we assigned disability weights of 0.254 (Infectious disease: post-acute consequences (fatigue, emotional lability, insomnia)) to acute HCV, chronic HCV, and chronic HCV under treatment. Weights of 1-(1-0.254)(1-0.194) (Infectious disease: post-acute consequences (fatigue, emotional lability, insomnia) and Decompensated cirrhosis of the liver) were assigned to chronic HCV with cirrhosis and chronic HCV with cirrhosis under treatment. Weights of 0.194 (Decompensated cirrhosis of the liver) were assigned to cirrhosis without chronic HCV. Weights of 1-(1-0.254)(1-0.508) (Infectious disease: post-acute consequences (fatigue, emotional lability, insomnia) and Terminal phase: with medication (for cancers, end-stage kidney or liver disease)) were assigned to HCC and LTX.

#### Statistical methods

##### Model fitting

We fitted the model to data described above to generate outcomes consistent with available data.

We ran the model from 1973 (the start of the injecting epidemic in Norway) to 2030. We used a limited-memory Broyden-Fletcher-Goldfarb-Shanno optimizer with bounded constraints (L-BFGS-B[11]) to find the transition parameters that gave the smallest weighted least-squares error when comparing model estimates to observed data.

The transition probability boundaries were specified using literature and data from Norwegian registries (Table 2). The yearly numbers of new PWIDs and relapse/cessation rates were provided by a separate estimation process (as detailed above), and these were hardcoded into the model.

After transition probabilities were calculated for each year, confidence intervals for each probability were calculated using a likelihood ratio test. Simply stated, the likelihood of the data for that year coming from a model with the calculated transition probabilities was calculated. Each transition probability was then walked to a minimum and maximum value, corresponding to the point where the likelihood ratio test became significant. A crude estimation of the standard error was then calculated by dividing the width of the confidence interval by 3.92 (2*1.96).

Subsequently, 1000 random draws of each transition probability were drawn from a random beta distribution, calibrated using the methods of moments. For each of these 1000 draws, the model was run and estimates for incidence, prevalence, YLLs, YLDs, and DALYs were calculated. The 2.5th and 97.5th percentiles were saved as the 95% confidence interval. Model fit to observed data was evaluated visually for each variable (Figure S3).

The model was implemented in R (software available at <https://www.r-project.org/>). Access to the full code (including source code for necessary additional packages/libraries) used to fit the model and subsequently generate all estimates, graphs, and tables can be obtained by contacting the corresponding author.
