# Supplementary material for: Modelling the burden of hepatitis C infection among people who inject drugs in Norway, 1973–2030
Source: BMC Infect Dis. 2017 Aug 3;17:541. doi: 10.1186/s12879-017-2631-2 (PMC5543437; doi:10.1186/s12879-017-2631-2)
Supplement: Supplementary file 3 — Table S1. Summary of model estimates from the compartmental model to estimate the burden of hepatitis C associated with people who inject drugs in Norway. (DOCX 21 kb) [file 12879_2017_2631_MOESM3_ESM.docx]

### Supplemental table

**Table S1**. Summary of model estimates from the compartmental model to estimate the burden of hepatitis C associated with people who inject drugs in Norway.

|  | **Total** | | | **PWID** | | | **Former PWID**  (will relapse) | | | **Former PWID**  (won’t relapse) | | |
| --- | --- | --- | --- | --- | --- | --- | --- | --- | --- | --- | --- | --- |
|  | **2000** | **2015** | **2030** | **2000** | **2015** | **2030** | **2000** | **2015** | **2030** | **2000** | **2015** | **2030** |
| ***Overlapping prevalence*** | |  |  |  |  |  |  |  |  |  |  |  |
| Alive | 17613 | 22292 | 23355 | 9108 | 8452 | 7824 | 5732 | 7989 | 7529 | 2773 | 5851 | 8002 |
| HCV+ (incl. treatment) | 7911 | 7682 | 5552 | 4441 | 3455 | 2328 | 2413 | 2609 | 1721 | 1057 | 1618 | 1503 |
| HCV+ (excl. treatment) | 7525 | 6892 | 4657 | 4259 | 3141 | 2011 | 2274 | 2321 | 1424 | 992 | 1429 | 1222 |
| HCV treatment | 387 | 790 | 895 | 183 | 314 | 317 | 140 | 287 | 297 | 64 | 189 | 281 |
| ***Discrete prevalence*** |  |  |  |  |  |  |  |  |  |  |  |  |
| HCV+ chronic | 6622 | 6549 | 4486 | 3438 | 2795 | 1793 | 2225 | 2341 | 1473 | 959 | 1414 | 1220 |
| Cirrhosis | 771 | 1421 | 1419 | 304 | 483 | 425 | 299 | 525 | 470 | 169 | 413 | 524 |
| HCC | 20 | 33 | 27 | 8 | 13 | 10 | 8 | 11 | 8 | 5 | 9 | 8 |
| Transplant | 1 | 10 | 13 | 0 | 1 | 1 | 1 | 5 | 6 | 0 | 4 | 7 |
| ***Incidence*** |  |  |  |  |  |  |  |  |  |  |  |  |
| HCV+ acute | 781 | 381 | 294 | 781 | 381 | 294 |  |  |  |  |  |  |
| HCV+ chronic | 605 | 285 | 219 | 521 | 245 | 189 | 69 | 33 | 25 | 15 | 7 | 5 |
| Cirrhosis | 86 | 88 | 58 | 42 | 36 | 22 | 31 | 32 | 19 | 13 | 19 | 16 |
| HCC | 13 | 24 | 23 | 5 | 8 | 7 | 5 | 9 | 7 | 3 | 7 | 8 |
| Transplant | 1 | 10 | 13 | 0 | 1 | 1 | 1 | 5 | 6 | 0 | 4 | 7 |
| ***Yearly mortality*** |  |  |  |  |  |  |  |  |  |  |  |  |
| Cirrhosis | 14 | 20 | 18 | 6 | 7 | 6 | 5 | 7 | 6 | 3 | 6 | 7 |
| HCC | 11 | 18 | 15 | 4 | 7 | 6 | 4 | 6 | 5 | 2 | 5 | 5 |
| Transplant | 0 | 1 | 2 | 0 | 0 | 0 | 0 | 1 | 1 | 0 | 1 | 1 |
| Total HCV related | 25 | 40 | 36 | 10 | 14 | 12 | 9 | 14 | 12 | 5 | 11 | 13 |
| Total not HCV related | 243 | 256 | 354 | 233 | 218 | 228 | 6 | 19 | 48 | 4 | 19 | 78 |
| ***Cumulative mortality*** |  |  |  |  |  |  |  |  |  |  |  |  |
| HCV related | 243 | 764 | 1345 | 107 | 308 | 508 | 92 | 286 | 484 | 43 | 170 | 354 |
| Not related to HCV | 3165 | 6973 | 11444 | 3075 | 6548 | 9872 | 62 | 250 | 736 | 28 | 175 | 836 |
| ***YLLs*** |  |  |  |  |  |  |  |  |  |  |  |  |
| Cirrhosis | 588 | 665 | 394 | 259 | 250 | 131 | 219 | 246 | 135 | 110 | 169 | 127 |
| HCC | 429 | 576 | 326 | 170 | 231 | 134 | 166 | 202 | 101 | 93 | 143 | 91 |
| Transplant | 7 | 47 | 51 | 1 | 3 | 3 | 4 | 24 | 24 | 2 | 19 | 24 |
| Total HCV related | 1024 | 1288 | 771 | 430 | 485 | 268 | 389 | 472 | 260 | 205 | 331 | 243 |
| Total not HCV related | 12231 | 9822 | 8730 | 11829 | 8807 | 7019 | 265 | 552 | 757 | 137 | 464 | 954 |
| ***YLDs*** |  |  |  |  |  |  |  |  |  |  |  |  |
| HCV+ acute | 198 | 97 | 75 | 198 | 97 | 75 | 0 | 0 | 0 | 0 | 0 | 0 |
| HCV+ chronic | 1682 | 1664 | 1139 | 873 | 710 | 455 | 565 | 595 | 374 | 244 | 359 | 310 |
| Cirrhosis | 250 | 423 | 428 | 103 | 148 | 130 | 95 | 154 | 140 | 52 | 120 | 158 |
| HCC | 13 | 21 | 17 | 5 | 8 | 7 | 5 | 7 | 5 | 3 | 6 | 5 |
| Transplant | 1 | 6 | 8 | 0 | 0 | 0 | 0 | 3 | 4 | 0 | 3 | 4 |
| Total HCV related | 2144 | 2210 | 1667 | 1180 | 964 | 667 | 665 | 759 | 523 | 299 | 487 | 477 |
| ***DALYs*** |  |  |  |  |  |  |  |  |  |  |  |  |
| HCV+ acute | 198 | 97 | 75 | 198 | 97 | 75 | 0 | 0 | 0 | 0 | 0 | 0 |
| HCV+ chronic | 1682 | 1664 | 1139 | 873 | 710 | 455 | 565 | 595 | 374 | 244 | 359 | 310 |
| Cirrhosis | 838 | 1088 | 822 | 362 | 398 | 261 | 314 | 400 | 275 | 162 | 289 | 285 |
| HCC | 442 | 597 | 343 | 175 | 239 | 141 | 171 | 209 | 106 | 96 | 149 | 96 |
| Transplant | 8 | 53 | 59 | 1 | 3 | 3 | 4 | 27 | 28 | 2 | 22 | 28 |
| Total HCV related | 3168 | 3498 | 2438 | 1610 | 1449 | 935 | 1054 | 1231 | 783 | 504 | 818 | 720 |

PWID: people who inject drugs, HCV: hepatitis C infection; HCC: hepatocellular carcinoma; YLDs: years lost of disabilities; YLLs: years of life lost; DALYs; disability adjusted life years **†**: Proportion that is attributable to active PWIDs.
